# Mitigating Fixation Artifacts in Spatial Transcriptomics: Methodological Insights from Human Brain Tissue

**DOI:** 10.1007/s12035-026-06033-1

**Published:** 2026-07-15

**Authors:** Kassandra Georges, Maiken Krogsbaek, Sara Newell Jensen, Thomas Damm Als, Kasper Thorsen, Johannes Rødbro Busch, Jytte Banner, Jens Randel Nyengaard

**Affiliations:** 1 Core Center for Molecular Morphology, Department of Clinical Medicine, Aarhus University, Aarhus, Denmark; 2 Department of Molecular Medicine, Aarhus University Hospital, Aarhus, Denmark; 3 Section of Forensic Pathology, Department of Forensic Medicine, University of Copenhagen, Copenhagen, Denmark; 4 Department of Pathology, Aarhus University Hospital, Aarhus, Denmark; 5 Present Address: Department of Biomedicine, Aarhus University, Aarhus, Denmark; 6 Present Address: Danish Pain Research Center, Department of Clinical Medicine, Aarhus University, Aarhus, Denmark

**Keywords:** Spatial transcriptomics, Brain bank, Fixed tissue, RNAscope, Target retrieval

## Abstract

Human brain bank material offers a great potential for studying a wide range of diseases from psychiatric to degenerative disorders, stroke, and pain. Historically, the primary limitation has been the technical usability of long-term formalin fixed and stored tissue samples with inadequate RNA quality. However, new and emerging, highly sensitive techniques now enable the study of the human transcriptome in autopsied brain material. This study aimed to develop a protocol for analysing RNA expression in two distinct cell populations within a spatial context from long-term formalin fixed human hypothalamus samples from the Danish SURVIVE study, using the GeoMx Digital Spatial Profiler from NanoString. RNAscope assays were used to optimize target retrieval, followed by evaluation with QuPath quantification to determine optimal RNA binding conditions. Optimal RNA availability was achieved with baking the slides at 60 °C for 1 h, followed by 30 min of 99 °C heat-induced target retrieval while maintaining tissue integrity. Subsequently, tissue was stained for either microglia or neurons with Iba1 and CRH specific antibodies and analysed in the GeoMx Digital Spatial Profiler. Whole transcriptome probes within the Iba1 and CRH segments were sequenced on the Illumina NovaSeq platform. The novel bioinformatic tool StandR was used for quality control and data analysis. The two cellular segments were compared, and we found 932 differentially expressed genes: 317 upregulated in the CRH segment, and 615 upregulated in the Iba1 segment. This paper presents a detailed workflow and highlights the ability to obtain relevant transcriptomic information from long-term fixed human brain tissue.

**Supplementary Information:**

The online version contains supplementary material available at 10.1007/s12035-026-06033-1.

## Introduction

Human brain bank collections offer great potential for studying a multitude of somatic and psychiatric disorders [1–4]. Various neurobiological techniques using post-mortem tissue have been developed over the last decade, leading to an increased interest in extracting data from brain bank tissue to elucidate complex psychiatric disorders [5]. One aspect of such techniques is gene profiling of symptomatically relevant regions of the brain [6]. Investigating the transcriptome in a spatial context allows for investigation of altered gene expression in the context of cellular and histological location as well as under different conditions [7]. There are now several types of techniques and multiple commercial options for performing spatial transcriptomics, which are either sequencing-based or imaging-based [7–9]. In sequencing-based methods, RNA is labelled either by placing tissue on a slide with an array of spatially barcoded probes (Visium standard, Visium HD, Slide-seq) or by hybridizing probes to the RNA in the tissue. The latter is subsequently imaged for spatial context and then microdissected (Tomo-seq) or segmented into regions from which the barcoded probes can be collected (GeoMx). The newest imaging-based methods such as Xenium (10X), CosMx (NanoString) and MERFISH (e.g. MERSCOPE from Vizgen) provide transcriptional information at a single-cell level based on iterative imaging of the tissue. While several methods are designed for fresh frozen tissue, both the Visium and GeoMx/CosMx platforms are compatible with formalin-fixed paraffin-embedded tissue (FFPE) [10, 11]. This compatibility makes it possible to work with archival tissue banks, where tissue has been preserved in formaldehyde or as FFPE for longer periods of time.

In this paper, NanoString’s GeoMx Digital Spatial Profiler (DSP) has been combined with RNA quality tests and advanced data analyses, with the aim of optimizing previously used methods of extracting genetic information from long-term fixed human brain. This was done by investigating the genetic profile of corticotropin-releasing hormone (CRH) positive neurons and ionized calcium-binding adaptor molecule 1 (Iba1) positive microglia in the paraventricular nucleus (PVN) of the human hypothalamus. The tissue used in this study has been stored in formaldehyde fixative for long periods of time, up to 800 days, leading to a range of technical challenges. State-of-the-art spatial profiling technologies, such as GeoMx DSP, require data analyses accounting for technical variability within the data, which previously used methods have been unable to do successfully [12]. Technical variations can occur within the slides during experiments, thereby creating a batch effect. This can lead to misinterpretation of the data, as the batch effect can mask the biological variation originating from the tissue [13]. Several other technical factors, including variability in tissue fixation and tissue preparation, become very relevant when working with tissue from brain banks, as they can contribute to unwanted sample-to-sample variations [12, 14]. A Bioconductor package, StandR, has been developed to limit these variations by providing optimized quality control, normalization, and assessment of GeoMx transcriptomics data through the incorporation of the standard linear modelling *limma-voom* differential expression pipeline rather than the traditional *T*-test for identifying differentially expressed genes (DEGs) [12].

In this paper, we aimed to investigate whether combining specialized antigen retrieval procedures with optimized data processing and statistical analysis could enhance the extracted transcriptomic information from autopsied hypothalami with highly varying fixation periods.

## Material and Methods

### Collection of Human Hypothalamus Tissue

The hypothalamus tissue was obtained from the Danish national forensic study, SURVIVE [15]. Oral or written consent was obtained from the relatives of the subjects for the use of material and clinical data for research purposes in compliance with national ethical standards. The SURVIVE study was approved by the Danish National Committee on Health Research Ethics (Protocol 1703611). Summary statistics of the subjects can be found in Supplementary Table 1.

The hypothalamus was dissected from the brain as a block of 4 × 4 × 4 cm and fixed in 4% neutral buffered formaldehyde (NBF) for 30 to 800 days. The tissue was afterwards cryoprotected for an average of 7 days in 25% sucrose and was then frozen in crushed carbon dioxide and stored at −80 °C. For each subject, either the left or right hemisphere of the hypothalamus was coronally sectioned in 10 µm sections on a cryostat (CryoStar Nx70, Epredia) with the blade holder at −12 °C and the specimen holder at −20 °C by systematic uniform sampling. Sections were mounted on SuperFrost Plus™ adhesion slides (Thermo Scientific) and stored at −80 °C. Immunohistochemistry (IHC) was subsequently performed on the tissue (see Supplementary File 1), and slides containing the paraventricular nucleus (PVN) were selected for further analysis.

### RNAscope

To assess the RNA accessibility of the hypothalamus tissue with varying fixation times, RNAscope in situ hybridization was performed using the RED protocol from ACD Bio [16] with adjustments to fixed frozen tissue incorporated [17]. For target retrieval, several different durations were tested to enhance the epitope availability, including 5, 15 and 30 min at 99 °C. The values are based on the ACD Bio protocol, where 5 min target retrieval at 99 °C was recommended for brain tissue. Proteinase K was applied for digestion. The housekeeping gene Ubiquitin C (UBC) was used for quality control, as it is highly expressed in brain tissue [18]. The positive probe for UBC (Hs-UBC-sense, 310,041, ACD Bio) was applied to all slides, except for one additional slide, where the negative probe (DapB, 310,043, ACD Bio) was applied.

The slides were mounted (EcoMount) and made ready to be scanned with Nanozoomer digital slide scanner (Hamamatsu) with a 20× lens (N.A. 0.75) and brightfield filter, which with digital enhancement in a 40× mode can provide a resolution of 0.23 μm/pixel, by use of the software Nanozoomer Digital Pathology-Scan (NDP.scan). Following imaging, probe binding of UBC in each section was quantified based on ACD Bio’s semi-quantitative evaluation. A score from 0 to 4 was assigned to the individual subject based on the average expression level per cell, as indicated by the number of probes visualized by red puncta localized in the cell (Supplementary Table 2). A qualitative evaluation of the staining was initially made, followed by an automatic quantification of the probes per cell by use of the bioimage analysis software QuPath. For the latter, regions of interests (ROIs), including areas from the PVN and from the arcuate nucleus (ARC), were assessed by the protocol from ACD Bio [19]. For every area, three ROIs were chosen, each covering 450 × 450 µm. The cell detection analysis and following subcellular spot detection had the following parameters: Cell detection: minimum area, 20 µm^2^; maximum area, 100 µm^2^; intensity threshold, 0.08. Subcellular spot detection: minimum spot size, 0.5 µm^2^; maximum spot size, 1.5 µm^2^; detection threshold, 0.15.

### Spatial Transcriptomics

To spatially profile the different cell types in the PVN, NanoString’s spatial transcriptomics method GeoMx Digital Spatial Profiler (DSP) was used. Two different cell types were investigated: (1) the corticotropin-releasing hormone (CRH)^+^ neurons and (2) the Iba1^+^ microglia. The experiment was performed following the standard NanoString protocol for fixed frozen (FxF) tissue for RNA assays [20] with some modifications (see overview in Table 1). When preparing the tissue samples, the protocol differed from the original protocol, as the brain tissue was baked for 60 min at 60 °C compared to the recommended 30 min. Secondly, during the sample preprocessing steps, target retrieval was tested at several temperatures and durations. In trial 1, the recommended 85 °C and 15 min duration for brain tissue was used, and in trial 2, 99 °C and 30 min duration was used. The slides were digested at 37 °C with Proteinase K at 0.1 µg/mL, compared to the 1 µg/mL as recommended in the original FxF protocol. For the in situ hybridization, the following concentrations were used for each slide: 200 μL Buffer R, 25 μL Atlas Probe Mix (Whole Transcriptome Atlas, GMX-RNA-NGS-HuWTA-4, HWTA21004, NanoString), 25 μL DEPC-treated H_2_O. For the morphology markers, CRH (Rabbit anti-CRH, T4037, BMA Biomedicals, 1:1000) and Iba1 (Rabbit anti-Iba1, 019–19741, Wako, 1:1000) antibodies diluted in buffer W were used alongside the nuclei marker SYTO83 (S11364, Invitrogen), compared to SYTO13 used in the original protocol to identify the cells. This change of nuclei dye was made due to the reduced autofluorescence in Cy3 in comparison to FITC, which SYTO13 originally was imaged in. The following secondary antibodies diluted in buffer W were used for CRH and Iba1, respectively: Alexa Fluor 594 (goat anti-Rabbit IgG, 1:400, A-11012, Invitrogen); Alexa Fluor 488 (goat anti-rabbit IgG, 1:400, A11034, Invitrogen). In between staining rounds, the slides were incubated with an IgG isotype control for 30 min.

**Table 1:** Standard and optimized protocol steps for GeoMx DSP for variable fixation times of human brain tissue[20]

	**NanoString standard (brain)**	**Optimized for long fixation**^**a**^
Baking at 60 °C	30 min	1 h
Target retrieval temperature	85 °C	99 °C
Target retrieval duration	15 min	30 min
Proteinase K digestion	1 µg/ml	0.1 µg/ml

^a^Fixation duration of 30 to 800 days in 10% NBF

The tissue was imaged on the GeoMx DSP instrument. CRH^+^ neurons were imaged with Texas red (615 nm, 300 ms exposure time), Iba1^+^ microglia with FITC (525 nm, 300 ms exposure time), and the nuclei stain SYTO83 with Cy3 (568 nm, 300 ms). Cy3 was used as the focus channel. Images of the sections were captured with a 20× objective (N.A. 0.45), and based on these, two to four ROIs with dimensions of maximum 660 µm × 785 µm were positioned along the PVN for each subject (Supplementary Fig. 1). Within every ROI, two sub-regions with biological targets, also known as area of illumination (AOI), were made; one for CRH^+^ neurons and one for Iba1^+^ microglia. The segment identifying CRH^+^ neurons and the segment identifying Iba1^+^ microglia had the following characteristics, respectively: Erode 1 µm, N-Dilate 2 µm, Hole Size 160 µm^2^, Particle size 15 µm^2^; and Erode 1 µm, N-Dilate 2 µm, Hole Size 1 µm^2^, Particle size 10 µm^2^. For the individual ROIs, the threshold sensitivity for each channel was modified to ensure coverage of all cells of interest. The barcodes from the CRH^+^ neuron segments were cleaved off and collected in a 96-well collection plate first, and the Iba1^+^ microglia segments second.

After collection, library preparation was performed according to the manufacturer’s protocol [20]. The collected DSP tag aspirate was thawed and dried on a thermocycler at 65 °C, then resuspended in 10 µL of nuclease-free water. A 4 µL aliquot of each resuspended aspirate was used for PCR amplification with NanoString SeqCode primers and PCR Master Mix (NanoString). The resulting PCR products were pooled and purified through two rounds of AMPure XP bead purification (Beckman Coulter, cat. no. A63882). Library quality control was conducted using a 4200 TapeStation (Agilent) and Qubit v2 (Fisher Scientific) before sequencing on a NovaSeq 6000 (Illumina). For the full protocol, see Supplementary File 2.

Additionally, to assess the IHC quality, the signal to noise ratio (SNR) was found for the ROIs of trial 1 and 2. SNR was calculated by measuring the mean fluorescence intensity of the positive staining in ImageJ for each of the three channels FITC, Cy3, and Texas Red (for Iba1, SYTO83, and CRH, respectively) compared to a negative staining from corresponding ROI. The mean of the ROIs for one subject was calculated and compared between trials. The nuclei count/AOI for the individual subjects in trial 1 and 2 is available in Supplementary Table 3. Additionally, raw reads/µm^2^ and AOI surface area for the corresponding sections are available in Supplementary Tables 4 and 5, respectively.

### Data Analysis—GeoMx NGS Pipeline and DSP Control Center

The generated FASTQ files were processed using the GeoMx NGS Pipeline v2.5.1 to produce output digital count conversion (DCC) files. In summary, raw reads were trimmed to remove adapter sequences and overlapping paired-end reads were merged. The resulting stitched reads were then aligned to barcodes in the reference assay, generating aligned reads. These aligned reads were used to assign raw counts to biological target names. PCR duplicates identified by matching barcodes in the unique molecular identifier (UMI) region were removed, resulting in deduplicated (unique) reads that were converted into digital counts.

The final DCC output files were uploaded to the DSP Control Center on the GeoMx DSP instrument. Within the DSP Control Center, the obtained digital counts were mapped back to their corresponding tissue locations. Quality control (QC) procedures were applied, including filtering out AOIs with insufficient raw reads, removing genes below the limit of detection (LoD), assessing negative probe performance, and ensuring a minimum cell count and surface area per AOI. For quality control comparison between trial 1 and 2, the FASTQ files were downsampled to the recommended 100 reads/µm^2^ before analysis in the DSP Control Center. Unique reads/µm^2^ were compared between samples and trials using a linear mixed-effects model (LMM) with trial as a fixed effect and subject as a random un-nested effect.

### Data Analysis—StandR

Data analysis was performed using StandR [12], a spatial transcriptomic analysis R-package for GeoMx DSP data [21]. First step of the QC was pre-processing of the probes, where low quality ROIs were excluded. The probe QC count was converted to log-transformed counts-per-million (logCPM). An expression threshold (*T*) was calculated using the logCPM data by taking the logarithm of the sum of a minimal count (*n*_min_) of 5 divided by the median library size (M(*L*)) and 2 divided by the mean of library size ($\overline{L }$), using the following formular:

$$T=\mathrm{log}\left(\frac{{n}_{\mathrm{m}\mathrm{i}\mathrm{n}}}{M\left(L\right)}+ \frac{2}{\overline{L}}\right)$$

Genes with a low expression were removed by calculating the Limit-Of-Quantification (LOQ) per segment, based on the distribution of k negative control probes in segment *i*, using this formula:

$$LO{Q}_{i}={\left(\prod\limits_{j=i}^{k}{x}_{ij}\right)}^\frac{1}{k}\times {\left[\mathrm{exp}\left(\sqrt{\frac{1}{k}{\left(\sum\limits_{j=1}^{k}\mathrm{ln}\,{x}_{ij}-\frac{1}{k} \sum\limits_{j=1}^{k}\mathrm{ln}\,{x}_{ij}\right)}^{2}}\right)\right]}^{n}$$

Two geometric standard deviations (*n* = 2) above the geometric mean of the negative probes were used as the LOQ.

Normalization and batch correction were performed using the Remove Unwanted Variation 4 approach (RUV4) [22], which uses negative control genes (NCGs) to remove unwanted variation. The top 300 NCGs were identified as the 300 least variable genes (ranked by coefficient of variation) across slides, assuming that batch effect is mostly due to slide effects. To evaluate the biological importance of the 300 NCGs and evaluate whether the set of 300 NCG genes exhibited greater pathway cohesion than expected by chance, we quantified their similarity to curated biological pathways using the Jaccard index and compared the observed value with an empirical null distribution generated by permutation. Gene sets were obtained from the MSigDB *Homo sapiens* C5 category and BP subcategory collection. Pathways were converted to lists of unique gene symbols, and only genes present in the study’s background universe were retained. The observed statistic was defined as the mean Jaccard similarity across all pathways:

$${J}_{obs}=\frac{1}{K}\sum_{k=1}^{K}J(G,{P}_{k})$$

where *K* is the number of pathways after filtering. *P*_*k*_ is the *k*th pathway and *G* is the NCG gene set. To assess whether the observed similarity exceeded that expected by random gene selection, we performed a permutation test. In each of *N* = 2000 permutations, we randomly sampled 300 genes without replacement from the 2968-background gene universe used in the Differential Expression (DE) analysis (see below), preserving the original sample size. For each permuted gene set we computed the mean Jaccard similarity across pathways, generating a null distribution ${\left\{{J}_{\mathrm{n}\mathrm{u}\mathrm{l}\mathrm{l},i}\right\}}_{i=1}^{N}$. This procedure therefore evaluates pathway cohesion under the constraint that randomly drawn genes originate from the same gene universe used for the NCG selection. The empirical one-sided *p*-value was calculated as:

$$p=\frac{1+{\sum}_{i=1}^{N}1*\left({J}_{\mathrm{n}\mathrm{u}\mathrm{l}\mathrm{l},i}\ge {J}_{\mathrm{o}\mathrm{b}\mathrm{s}}\right)}{N+1}$$

which tests whether the observed mean similarity is greater than expected by chance. A high *p*-value indicates that the NCG set does not display unusually high pathway similarity relative to random gene sets of the same size. A *p* = 0.68 was obtained.

To further evaluate whether the 300 NCG genes exhibited pathway-level clustering, we performed an overrepresentation analysis using curated Gene Ontology Biological Process (C5:BP) gene sets from MSigDB. Gene sets were obtained with msigdbr (species = *Homo sapiens*, category = “C5”, subcategory = “BP”) and converted into a list mapping each pathway to its constituent gene symbols. Only genes present in the current study’s background universe (*n* = 2968 genes, see below) used in the DE analysis (see below) were retained. All gene symbols (NCGs and background genes) were converted to Entrez IDs using org.Hs.eg.db. Pathway annotations were formatted into a TERM2GENE table compatible with clusterProfiler::enricher(). Overrepresentation analysis was performed on the NCG gene set relative to the filtered universe, and the number of significantly enriched pathways (*q* < 0.05, Benjamini–Hochberg correction) was recorded. None of the nominally significant pathways remained significant after correction for multiple testing. To assess statistical significance, a permutation test was performed. In each of 1000 iterations, 300 genes were randomly sampled from the 2968-gene universe, and the number of significant pathways was recalculated. An empirical one-sided *p*-value was then computed as the fraction of permutations in which the number of significant pathways was greater than or equal to that observed for the NCGs. This procedure accounts for the specific background universe and provides a robust null model for pathway cohesion. A permutation p-value of $p\approx 1$ was obtained.

Additionally, we performed RUV4 batch correction using the next batch of 300 least variable genes (301:600) as NCGs, and repeated downstream analyses, to ensure that the downstream differential expression analysis and gene set enrichment analysis did not change substantially.

Using the identified NCGs and specifying the variable of biological interest, the RUV4 method was run for *k* = 1 to *k* = 5, where *k* is the number of unwanted factors to correct for. Principal Component Analysis (PCA) was subsequently used for evaluating the performance of the RUV4 approach for each value of *k*, by constructing multiple PCA plots with colour schemes based on different variables, i.e. the variable of biological relevance and several potential confounding variables (slide name, segment, body mass index (BMI), age, section, post-mortem interval (PMI) (hr), fixation period (days), fixation period group (<100 days, 100–365 days, >365 days). These covariates are used in the RUV4 correction and based on visual inspection of PCA plots for *k* = 0 through *k* = 5, coloured by two biologically relevant variables and six potential confounding variables (see Supplementary Fig. 5), a value of *k* = 3 was chosen as the optimal number of unwanted factors for the RUV4 approach. This ensured effective correction of potential confounding variables, while keeping as much of the biological variation of interest as possible. In addition, relative log expression (RLE) plots of normalized counts were used to assess the performance of the batch correction. For comparison, the batch removal approach implemented in the limma R-package [23] was also applied. Based on PCA plots, six summary statistics (adjusted rand index, Jaccard similarity coefficient, silhouette coefficient, chi-squared coefficient, Mirkin distance and overlap coefficient [12]) that measured the effectiveness of batch correction for RUV4 (*k* = 3) and limma, we concluded that RUV4 with *k* = 3 was most effective in batch correction, while preserving as much of the biological variation of interest as possible.

To ensure AOIs corresponded to the cell type of interest, the segments for CRH or Iba1 were investigated for the expression of CRH and CD74, respectively. With cell type as the fixed effect, differential expression analysis between CRH and Iba1 segments within all subjects was performed using the *limma-voom* approach on the log-CPM values and with the weight matrix generated using the RUV4 approach with k = 3 covariates, corresponding to this model [24]:

$${{\boldsymbol{Y}}}_{{\boldsymbol{g}}}={\boldsymbol{X}}{{\boldsymbol{\beta}}}_{{\boldsymbol{g}}}+{{\boldsymbol{W}}}_{1}+{{\boldsymbol{W}}}_{2}+{{\boldsymbol{W}}}_{3}+{{\boldsymbol{\rho}}}_{{\boldsymbol{g}}}+{\boldsymbol{\epsilon}}$$

where *Y*_*g*_ is gene expression measurements for a gene, $X{\beta}_{g}$ is the biological effects of gene *g*, *W*_*1*_, *W*_*2*_ and *W*_*3*_ are RUV unwanted-variation factors, ${\rho}_{g}=cor({y}_{gij},{y}_{gij})$ is the intra and inter individual correlation for gene *g* and ${\boldsymbol{\epsilon}}$ is residual noise.

Intra- and inter-individual correlations were taken into account, by using the *duplicateCorrelation* function with subject/patient ID as the block factor and passing the results to the *lmFit* function. Both functions are from the limma R-package. Thus, for each gene we fit a weighted linear model in limma, using voom-derived precision weights and a fixed-effects design comprising the primary covariate and K = 3 RUV components, while accounting for within-patient correlation through duplicateCorrelation (two-step estimation) and block-correlated Generalized Least Squares (GLS). Differentially expressed genes (DEGs) were defined based on *p*-values (at the *p* = 0.05 significance level) adjusted for multiple testing across genes, using the Benjamini and Hochberg approach [25]. For the NCGs, the RUV4 batch correction for the 600 least variable genes (1:300 and 301:600) resulted in a Pearson’s correlation coefficient of log-Fold-Change (*r* = 0.99970, 95% CI: [0.99967; 0.99972], *p*-value < 2.2e-16) and -log_10_(P) (*r* = 0.9982, 95% CI: [0.9980; 0.9983], *p*-value < 2.2e-16) from the Differential Expression analyses indicate that results are similar for the two datasets with different NCGs for RUV4 batch correction.

Gene set enrichment analysis (GSEA) was subsequently performed in RStudio, using the Molecular Signatures Database (MSigDB) package from Bioconductor. The fold change between the genes was calculated and analysed with the gene set “GO BP—biological processes” from the gene ontology database, and the normalized enrichment score (NES) was calculated. The RUV4 batch correction for the 600 least variable genes (1:300 and 301:600) resulted in a Pearson’s correlation coefficient of NES (*r* = 0.9883, 95% CI: [0.9877; 0.9888], *p*-value < 2.2e-16) and -log_10_(P) (r = 0.9959, 95% CI: [0.9957; 0.9961], *p*-value < 2.2e-16) from the GSEA, indicating that the results were similar despite using two non-overlapping NCGs for RUV4 batch correction.

## Results

### RNA Accessibility Assessment

**Fig. 1:**
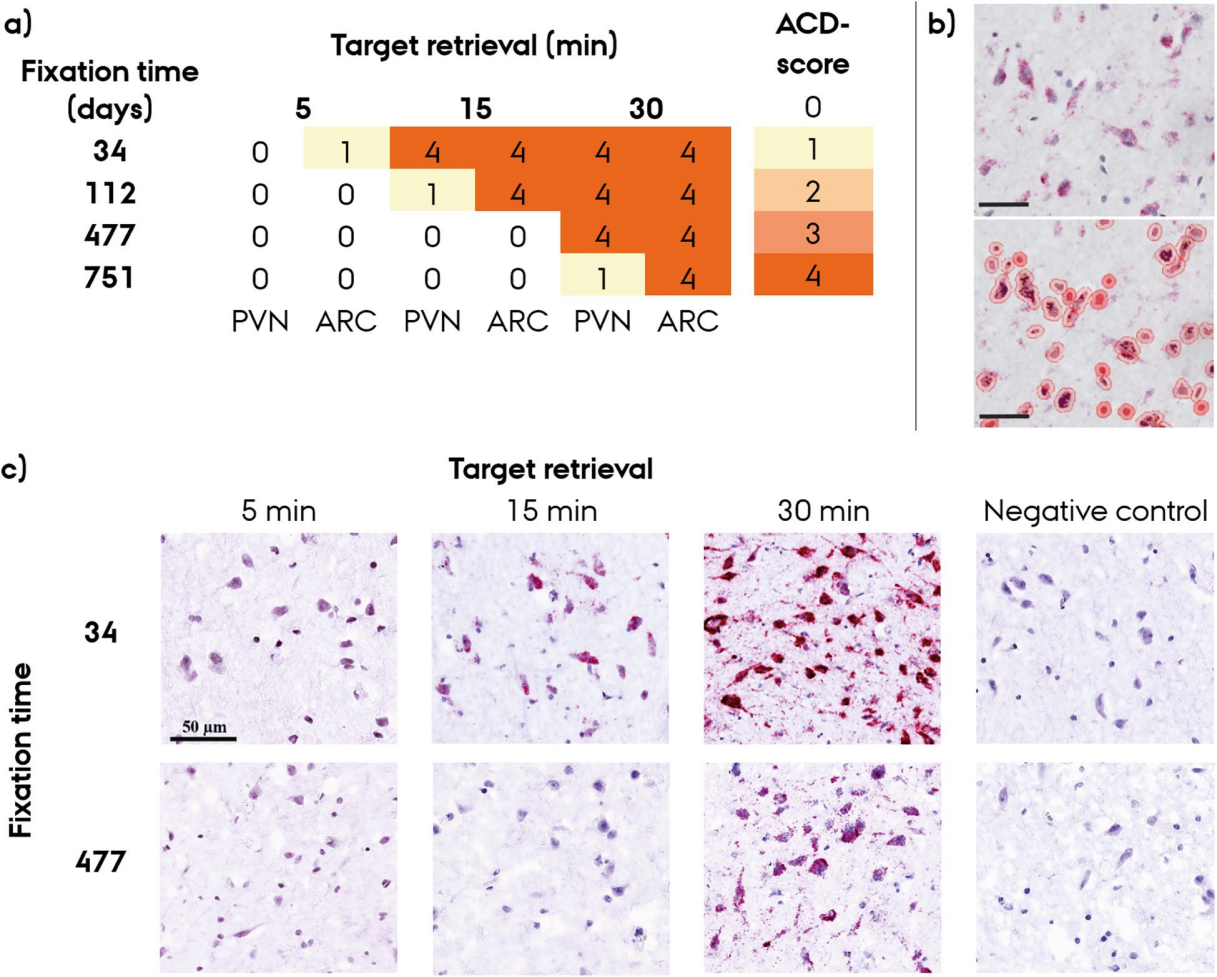
Comparison of RNAscope assay between ubiquitin C (UBC) expression in tissue with varying fixation times and with different target retrieval durations in the hypothalamic paraventricular nucleus (PVN) and the arcuate nucleus (ARC). **a** Table of QuPath quantified ACD scores showing the result of varying target retrieval durations in tissue with short and long fixation times. **b** Example of RNAscope image (top) and QuPath mask used for quantification (bottom) in the PVN. **c** RNAscope image examples of UBC expression in tissues with varying fixation times exposed to different durations of target retrieval (5, 15 and 30 min) in the PVN. Negative control: representative images of DapB expression (negative control gene, 15 min target retrieval). paraventricular nucleus, PVN; arcuate nucleus, ARC. Scalebars: 50 µm

To investigate the accessibility of the RNA in the tissue based on different fixation periods, RNAscope analysis was performed on PVN and ARC. When comparing the UBC expression in the tissue with varying fixation time, differences were observed depending on the duration of target retrieval used, as shown in Fig. 1. When the sections were exposed to 5 min of target retrieval at 99 °C, as recommended by the original protocol [17], no probe binding was observed in the PVN of either subjects with a short fixation period (<200 days) or subjects with a long fixation period (>200 days) (ACD score = 0). The same observation was made in the ARC, with one exception from the subject with the shortest fixation period (34 days) resulting in little UBC expression (ACD score = 1). Additionally, with this target retrieval duration, most of the sections incubated with the positive probe looked like the control sections receiving the negative probe (ACD score = 0). When increasing the target retrieval duration from 5 to 15 min, the tissue with the short fixation period (34 days) had several positive puncta, representing more available RNA with this treatment in both the PVN and the ARC (ACD score = 4). Interestingly, there was a large variation in visible puncta between the two different regions within the same section from the tissue that was fixed for 112 days, with the ARC scoring higher (ACD score = 1 in PVN and 4 in ARC). Contrarily, sections with a fixation period >400 days continued to show no UBC gene expression (ACD score = 0). Lastly, the target retrieval duration was increased to 30 min, and all the sections showed a gene detection with an ACD score > 1. Within the PVN, tissue with a fixation period < 500 days resulted in the highest score (ACD score = 4), whereas the tissue with a fixation period of 751 days showed less expression than the other fixation periods (ACD score = 1); however, it showed more expression with this treatment compared to shorter durations of target retrieval. Notably, independent of the duration of the fixation period, the ARC received the highest score throughout all the different sections. A manual evaluation was also made (Supplementary Fig. 2). The comparison between the manual and QuPath-generated ACD score is illustrated in Supplementary Fig. 3, which showed a comparable evaluation between the two types.

### Staining Quality with Different Target Retrieval Procedures

**Fig. 2:**
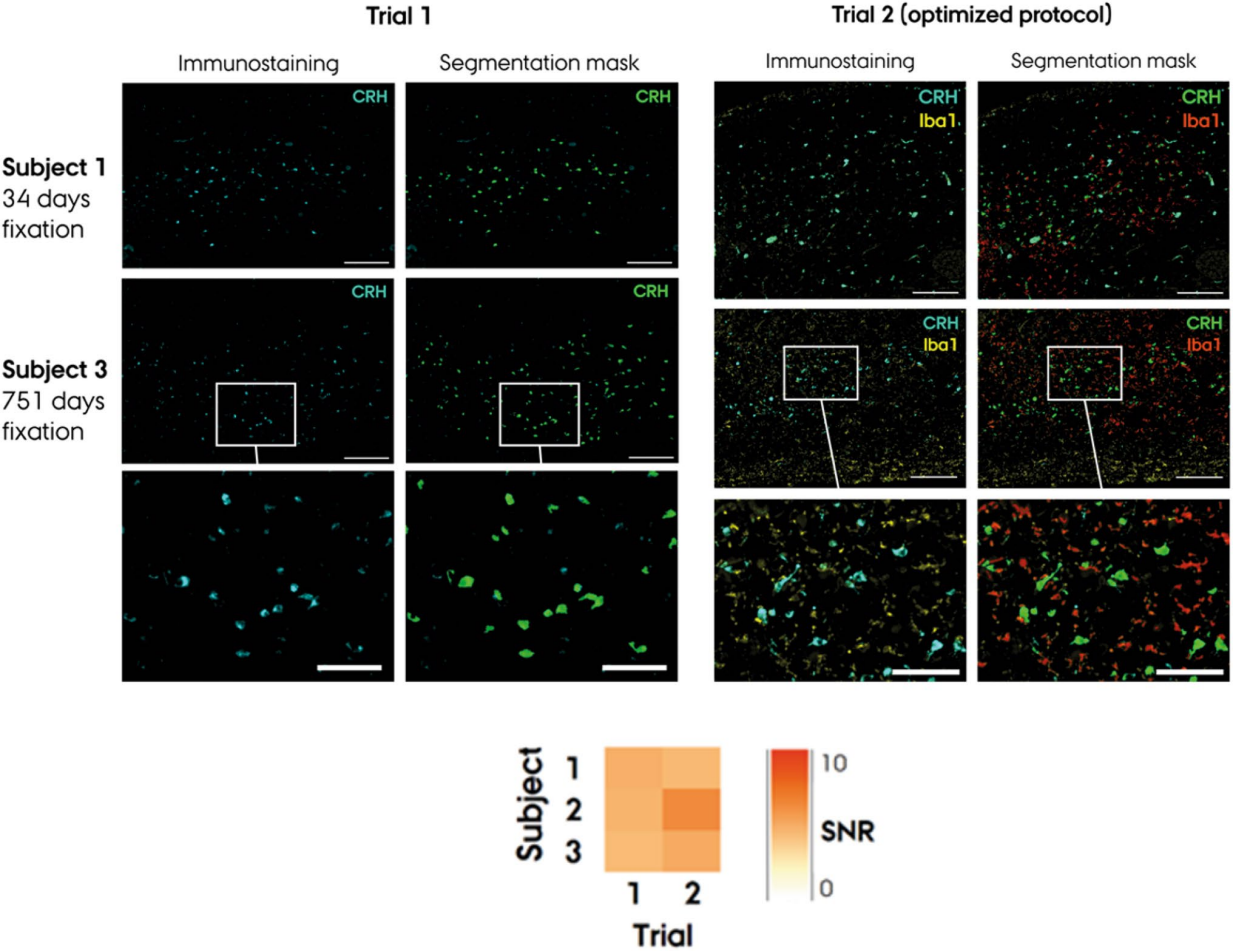
Staining quality through different target retrieval procedures: Top: Immunostaining and segmentation from trial 1 (left) and 2 (right) as visualized in GeoMx Digital Spatial Profiler prior to extraction and sequencing. Bottom: The signal to noise ratio (SNR) calculated for three subjects shows highly similar SNR values between trial setups. The fixation time (days) for subjects 1–3, respectively: 34, 477, 751. Scalebars: 250 µm (top 4), 100 µm (bottom 2)

To assess the staining quality of CRH between trial 1 and 2, which had received 15 min at 85 °C and 30 min at 99 °C, respectively, the SNR was calculated and visualized for three subjects that had been fixed for 34, 477, and 751 days, as shown in Fig. 2. The heatmap shows that independent of fixation duration of the individual subject and the target retrieval procedure, a similar SNR was calculated. The range of SNR for trial 1 was 4.4–5.8 (mean = 5.1), and the range for trial 2 was 4.5–7.4 (mean = 5.8). No SNR value was below 3, which is indicated by NanoString as a sufficient SNR [26].

### GeoMx Data Quality with Different Target Retrieval Procedures

**Fig. 3:**
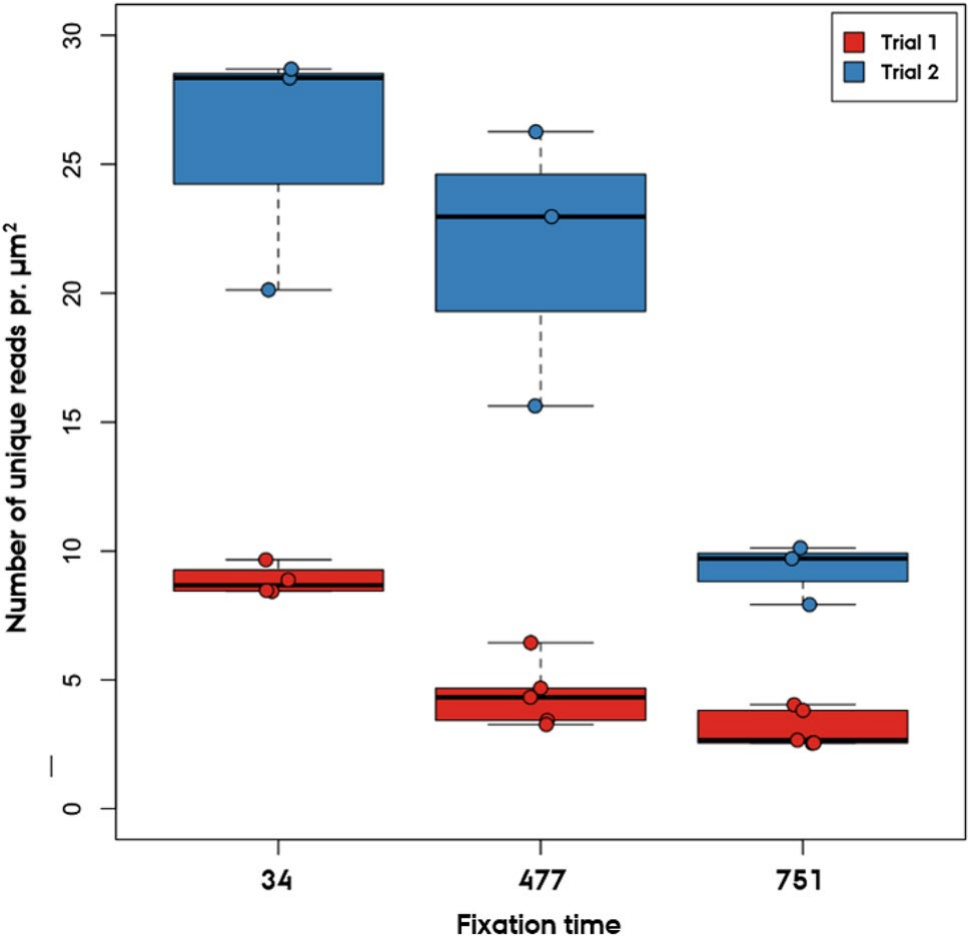
Boxplot showing the number of unique reads per square micron for the CRH segments in trial 1 and trial 2 for three patients. The linear mixed-effects model revealed a significant difference between the two treatments in the different trials (*p* < 0.001). Fixation time in days

The GeoMx sequencing libraries from trials 1 and 2 showed comparable fragment sizes and concentrations (203 bp and 3.5 nM vs. 201 bp and 4.5 nM), indicating similar library preparation quality. Despite this, the on-instrument QC pipeline revealed substantial differences between trial 1 and 2. To ensure consistency, the data were down-sampled to meet the recommended threshold of 100 reads/µm^2^ for both trials. After removing duplicated reads, the QC results demonstrated significantly more unique reads/µm^2^ in trial 2 compared to trial 1 (LMM: *p* = 9.11e-8), as shown in Fig. 3. Furthermore, for all sections, longer fixation times are associated with fewer unique reads/µm^2^ (Fig. 3).

### GeoMx Digital Spatial Profiling Analysis with StandR

The GeoMx data was analysed using the StandR pipeline and data analysis QC parameters are visualized in Supplementary Fig. 4. The minimum recommended number of cells from NanoString (100), was not achievable to reach due to small AOI area, limiting inclusion of the scattered CRH^+^ neurons over a large area in the PVN, as well as due to the variability in Iba1^+^ cells present between individuals. Gene QC is illustrated as a histogram distribution of the frequency of lowly expressed genes in each ROI in Supplementary Fig. 4-A. Based on the gene QC, genes with an expression < LOQ in 2% or more of the segments were removed, resulting in 2938 genes used in subsequent analysis. Secondly, ROI QC level was assessed, and the relationship between library size and cell count and AOI size is illustrated in frequency histograms in Supplementary Fig. 4-B and 4-C, respectively. After applying a threshold of five cells, 11 ROIs were excluded from the dataset due to subthreshold values. Additionally, PCA was performed on the data to visualise either biological or technical systemic variations in the data. Supplementary Fig. 4-D shows the variance explained by each component in the PCA.

**Fig. 4:**
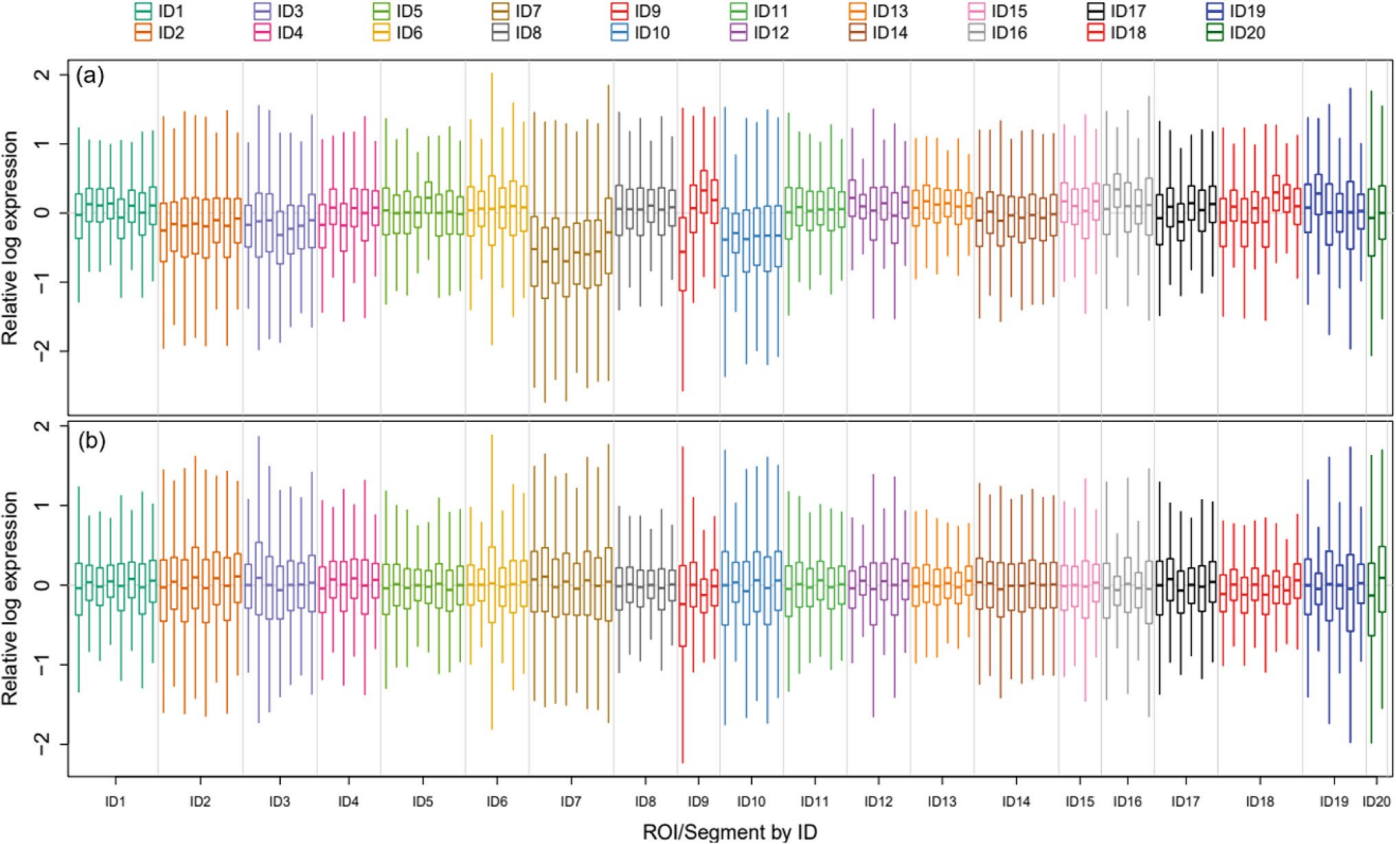
Relative log expression (RLE) plots for 20 subjects **a** before and **b** after RUV4 batch correction as boxplots by combination of ROI and Segment and colour coded at subject level

**Fig. 5:**
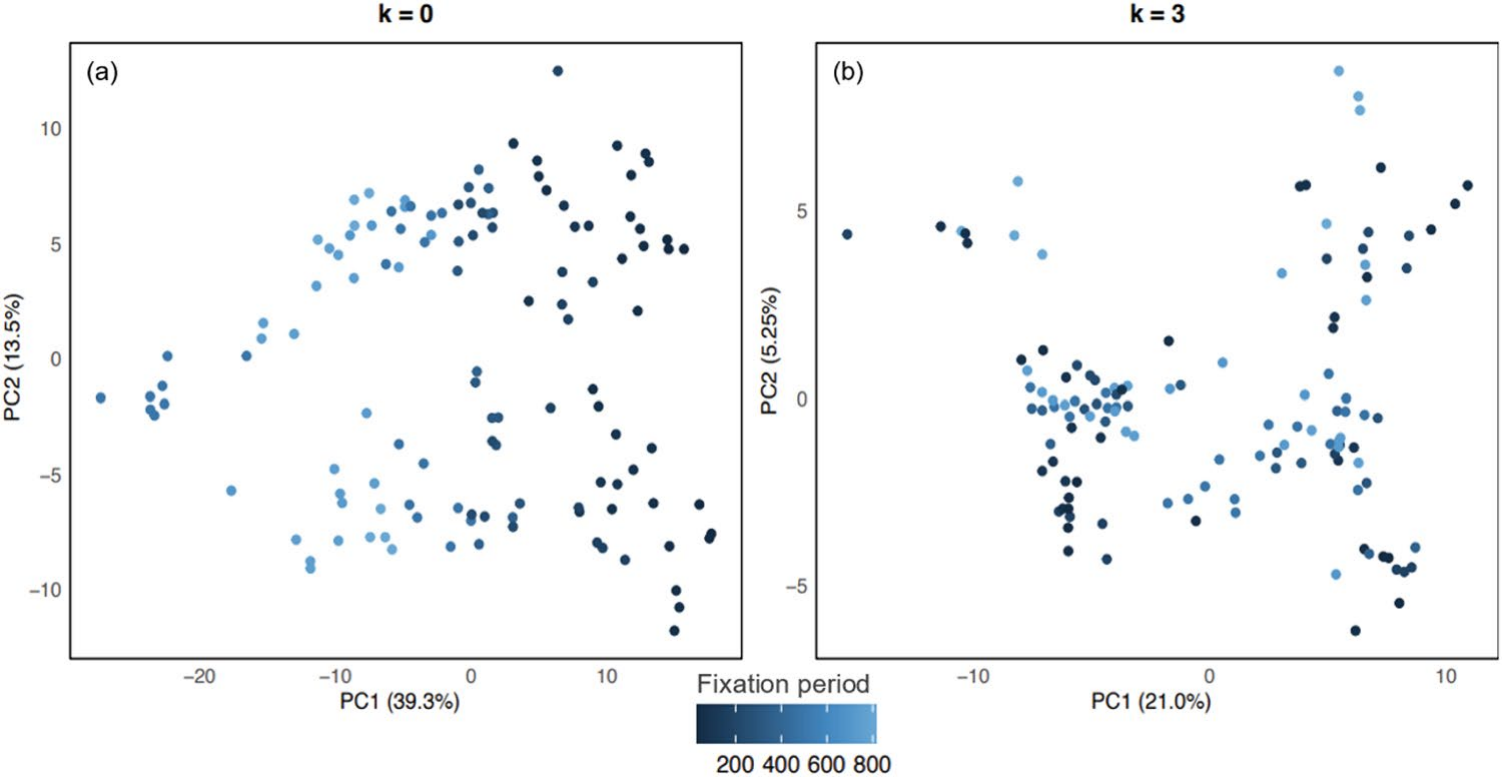
PCA showing RUV4 corrections with number of unwanted factors (k) correcting the fixation period. **a** *k* = 0 and **b** *k* = 3. Fixation period measured in days

To rectify technical variations observed in the QC steps, a normalization of the data was performed. The relative log expression (RLE) plots from before and after normalization are shown in Fig. 4. To compare data across ROIs from different sections and slides, batch correction was performed using the RUV4. For this correction method, the number of unwanted factors (k) was tested to optimally remove technical variation, including fixation period, between the sections. PCA of fixation period for number of unwanted factors 0 and 3 are illustrated in Fig. 5. PCA plots before and after the batch correction with *k* = 0 to *k* = 5 are illustrated in Supplementary Fig. 5, including factors such as biological target, slide, BMI, age, section, PMI, fixed period, and grouped fixation time.

### Analysis of Differentially Expressed Genes and Involved Gene Sets

**Fig. 6:**
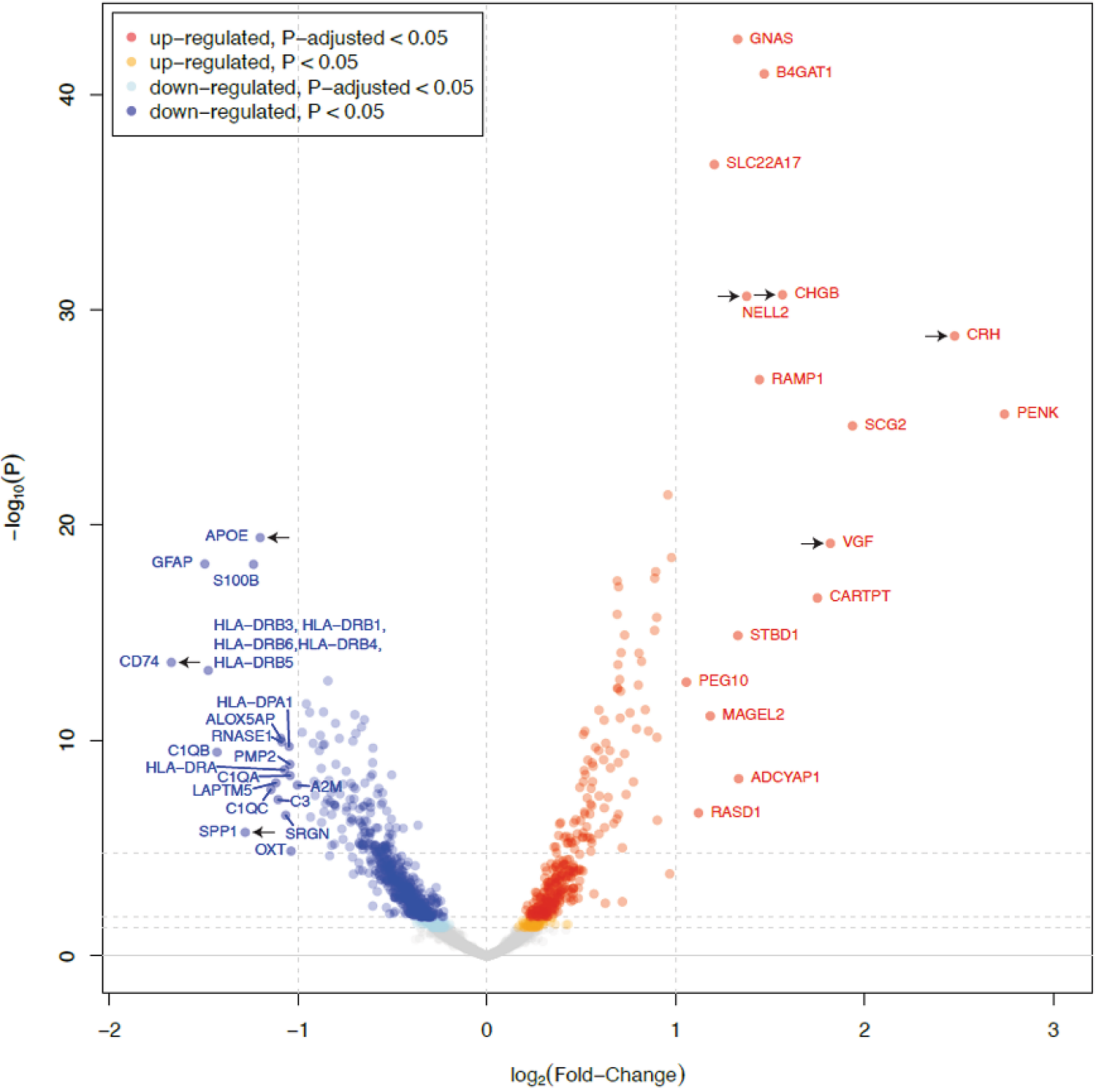
Volcano plot illustrating significantly differentially expressed genes based on the StandR analysis comparison of the genes increased (red) and decreased (blue) in the CRH segment. Data is based on the adjusted *p*-value. Marked by a black arrow are genes particularly stressing the differences in genetic profiles. Grey points are non-significant

Differentially expressed genes (DEGs) analysis comparing hypothalamic CRH segment to the Iba1 segment identified 932 DEGs: 317 upregulated and 615 downregulated in CRH, corresponding to 615 upregulated DEGs in Iba1. DEGs are visualized in the volcano plot in Fig. 6. From the data, it is possible to see that expression of the immune system-related genes APOE, CD74, and SPP1 are highly expressed in the Iba1 segment compared to the CRH segment, where CRH expression is significantly higher. Additionally, genes involved in neuronal activity, such as CHGB, NELL, and VGF are seen upregulated in the CRH segments. These genes are marked with arrows in the volcano plot (Fig. 6).

**Fig. 7:**
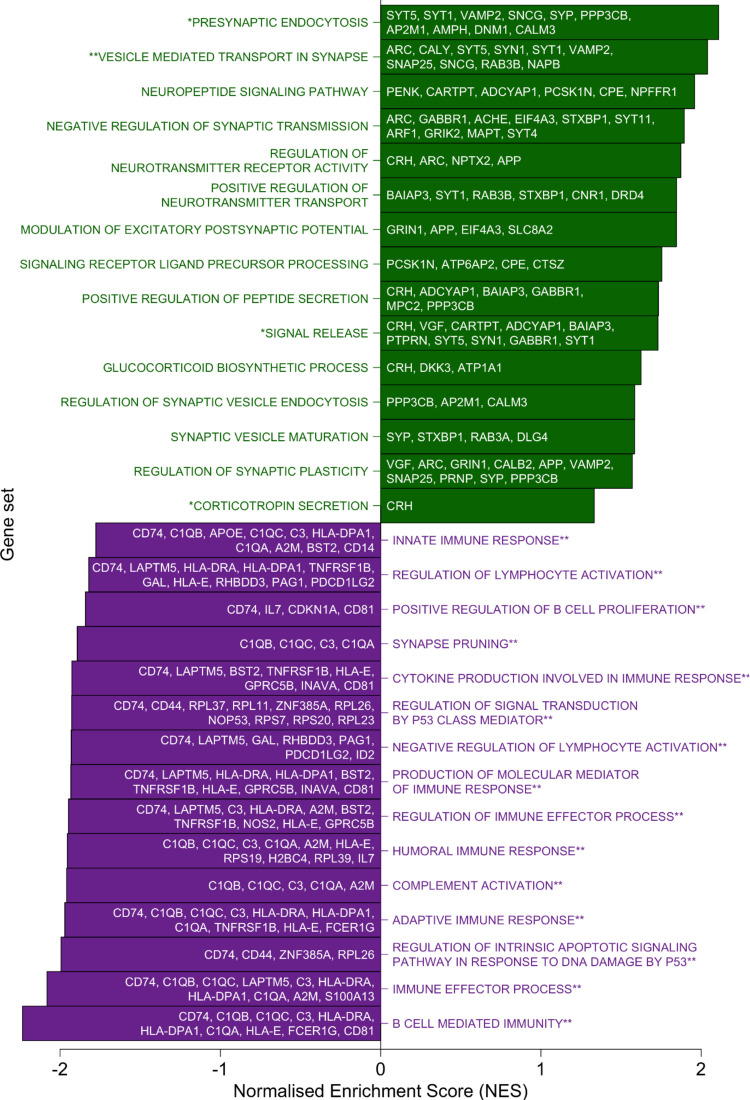
Gene set enrichment analysis (GSEA) comparing differentially expressed genes (DEGs) in CRH^+^ neurons (green) to Iba1^+^ microglia (purple) based on gene ontology biological processes. Fifteen gene sets for each cell type have been plotted based on their normalised enrichment score (NES). Genes in the bar indicate the main genes leading the enrichment. **P_adjust_ < 0.05, *P_adjust_ < 0.1

A gene set enrichment analysis (GSEA) was performed comparing the significant DEGs between CRH^+^ neurons and Iba1^+^ microglia. The analysis showed a clear separation between the expression pattern of the two different cell types. To illustrate the differences in their genetic profiles, 15 gene sets upregulated in each group were selected and their normalized enrichment scores (NES) are visualised in Fig. 7. The genes mainly upregulated in the CRH^+^ neurons were involved in synaptic transmission and plasticity, dictated by the genes SYT5, SYT1, VAMP2, SYP, SNAP25, and ARC, as well as neuropeptide regulation, shown by CRH, PENK, and CARTPT (*p* < 0.05, Fig. 7). Contrarily, the genes significantly upregulated in the Iba1^+^ microglia were mainly involved in pathways regulating the immune response, dictated by the genes CD74, IL7, HLA-DRA, RPS6, CD81, and CDKN1A, and in synaptic pruning, shown by the genes C1QB, C1QC, C3, C1QA (*p* < 0.05, Fig. 7).

## Discussion

We sought to optimize methods of extracting genetic information from long-term fixed human brain tissue by combining RNA quality analyses, improved antigen retrieval, cutting-edge spatial transcriptomics technology, and advanced data analyses. Tissue that has been fixed for a varying range of days (30 to 800 days) presents several biological and technical challenges. RNAscope analysis showed that increasing both the target retrieval duration and temperature could enhance RNA availability regardless of fixation duration. The data showed a clear tendency for the tissue that had been fixed the longest also required the longest target retrieval duration. One potential concern when increasing target retrieval intensity, and thereby subjecting the tissue to harsher conditions, is whether both the tissue and the antibody targets for subsequent staining can withstand the treatment. The data showed that independent of fixation duration of the individual subject, the IHC SNR was similar between the two trials of different target retrieval procedures. The overall SNR > 3 across the trials for the individual subjects indicated that the tissue and antibody binding can endure such treatment. Technical issues arising during library preparation or sequencing, as mentioned above, were similarly corrected during data analysis. Target retrieval had an important effect on the number of unique reads obtained from the AOIs, which was increased significantly with the optimized protocol.

When incorporating the novel data analysis, StandR, an extensive amount of genetic information was extracted from the highly fixed brain tissue. The performed quality control showed that technical variation which may have been present in the dataset was corrected for following filtering and normalization. Additionally, variation such as fixation duration could be corrected for without masking the biological variations between the two cell types. To further support the distinction between the two targets, a GSEA was performed in addition to the DEGs analysis. Both analyses showed that significantly upregulated genes found in the CRH^+^ neurons were involved in synaptic transmission and neuronal activity, whereas the Iba1^+^ microglia showed significantly increased gene expression highly involved in the immune system and inflammation, as expected. The data showed the specificity in the biological patterns, once more stressing the distinction in genes extracted from the highly fixed hypothalamic tissue.

We showed that it is possible to extract information from brain tissue that has been formaldehyde fixed for over 2 years. This could potentially allow for further post-mortem investigation of the aetiology of somatic and psychiatric disorders as well as discovering new diagnostic targets [27]. Fixation of tissue with formaldehyde has several advantages, as it preserves the architecture of the cells and prevents tissue degradation [28] allowing for the investigation of tissue donated to brain banks several years ago. However, a key challenge following formaldehyde fixation is the formation of intermolecular cross-links between formaldehyde and macromolecules [29]. This leads to an antigen masking resulting in decreased immunoreactivity [29]. Fortunately, methods such as heat-induced antigen retrieval have been developed to improve antigen detection by reversing the conformational changes following fixation [30, 31]. As there is no universal protocol of antigen retrieval covering these variations [31], it becomes an important parameter to consider, when pursuing data extraction from brain banks, where individual tissue samples can vary in post-mortem fixation treatment. An additional challenge with brain bank tissue is that it has traditionally been stored in formaldehyde for extended periods [32]. Studies have shown that this type of storage for longer than 6 months has a high DNA degradation [33], whereas storage at −80 °C as a method of storage can keep high RNA quality up to 23 years [34]. Here we show that fixed frozen methods of fixation allow for RNA investigation of tissue stored in formaldehyde for 800 days. Previous studies have shown that prolonged fixation time affects the RNA quality [35], but to which extent is debated still [36, 37]. Our data suggests that it is possible to extract enhanced RNA information by improving the accessibility of RNA by use of heat-induced target retrieval independent of fixation time. Other studies have tried to increase the heat-induced target retrieval duration from 15 to 25 min and showed an improvement in mRNA accessibility in over-fixed-tissue; however, it was suggested that an increase in detection may be due to increased unspecific binding [36] rather than due to increased accessibility. The same study, however, showed that the negative probe additionally increased in expression, leading to their interpretation of unspecific binding. The negative probe used in this study, contrarily, did not alter according to target retrieval duration, therefore contradicting the argument that the observed increased RNA accessibility is due to unspecific binding. Furthermore, when comparing the SNR between the morphology markers of the different treatments, no altered autofluorescence was observed, further supporting the notion of specific binding. Additionally, the improved bioinformatic pipeline used here allowed us to correct for potential variations occurring due to the prolonged fixation time between subjects. This becomes very relevant for future use of brain banks, where the fixation similarly can vary in duration between patients [38]. The StandR workflow further applies the *limma-voom* pipeline when analysing DEGs, instead of the traditional paired T-test, which is known to be less sensitive to specific outliers by abstaining genes as independent, allowing for a more precise estimation of biological variation [12, 23]. This approach was quite useful in this study, as the sample size of the cohort was relatively small and therefore may have been highly influenced by outliers.

The sample size is of course important to consider, as only three subjects of varying fixation duration were used in the first trial and 20 subjects in the second trial. While the small sample size in the first trial is a limitation in this study, it is evident that the number of unique reads per µm^2^ is decreased with increasing duration of fixation time. Nonetheless, the significant differences between the unique reads in the subjects between trial one and two are remarkable. Expectedly, the subject with the shortest fixation time showed the largest number of unique reads per µm^2^. While the subject with the longest fixation time showed a small increase in the second trial compared to the other subjects, the observed threefold increase in the second trial is still evident. The largest change, however, was observed in the patient with a fixation time of 477 days, which supports a futuristic approach to limiting the loss of genetic information in highly fixed tissue. Additionally, when using the *limma-voom* pipeline for correction, which gave a more precise estimation of the biological pattern, a clear distinction was observed between the different cell types, allowing us to investigate them further. The genes and associated pathways were fitting with the responsible cell types, as expected, which stresses the proof of concept of this experiment. While most upregulated genes in the microglia segment were related to immune responses, we did observe genes related to astrocytes, suggesting that the GeoMx mask covering Iba1+ microglia most likely also caught some astrocyte processes in the microglia proximity. We interpret the Iba1 segments as microglia enriched, due to the high presence of several established microglia-related genes, including CD74, APOE, SPP1, HLA-DRB1 and the complement genes C1QA, C1QB, C1Qc, and C3, in the Iba1 segment. The segment also showed increased expression of GFAP, SLC1A3 and GLUL, all genes involved in astrocytic function [39]. However, the following genes from the same category were not observed in our data: GLUT1, PC, GP, PKM2, LDH5, MCT4, FABP7, 3PGDH, SLC1A5, EAAT1, EAAT2, and AQP4, among others. Due to the absence of several genes highly involved in astrocytic processes as well as the presence of reactive glia-related genes, we hypothesize that the observed pattern reflects a partial astroglia/reactive signature, rather than a mixture of microglia and astrocytes. Several technical issues, including batch effect and fixation duration were corrected, as discussed above; nonetheless, one technical issue we could not overcome, was the sparsity of the cells of interest within these subjects. The individual ROI of the GeoMx DSP has a relatively small diameter when looking at cells that are scarcely distributed in the tissue. This required several ROIs from each subject within the same PVN section. Even then, the cell count for each AOI did not reach the recommended cell count per AOI according to NanoString’s recommendations, which may limit the statistical power of the study. However, while NanoString recommends including at least 100 cells per AOI, the relatively low number of cells in some AOIs may not pose a major issue for high expressors and may provide sufficient signal despite their limited abundance [40]. It is furthermore relevant to comment on our use of the housekeeping gene, UBC. Because of its abundant and stable expression in all tissue, it was used as an indicator of RNA preservation across fixation durations. This results in a reliable comparison between the effect of prolonged fixation durations on the transcripts, and how these effects may be reversed by improving target retrieval procedures. The robust UBC signal observed under these conditions confirmed that RNA was sufficiently preserved and accessible for downstream GeoMx analysis; however, it is still important to consult with the GeoMx DSP platform’s internal quality control measures to reliably interpret the data. The platform provides QC metrics (including negative controls, probe detection efficiency, and signal-to-noise thresholds) that independently verify successful RNA hybridization and detection across a broad dynamic range of transcripts within each sample, which is necessary when working with fixed tissue such as in this study. For future research into the genome of highly fixed cell types, however, it would be interesting to test out different types of housekeeping genes in regard to their expression pattern, particularly with a focus on low abundance, less stable transcripts.

## Conclusion

In conclusion, our optimized protocol combining an RNA accessibility test with the enhanced target retrieval protocol and the novel data analysis allowed us to extract relevant and reliable spatial transcriptomic data from brains that had been stored in formaldehyde for up to 800 days. This knowledge is relevant to further enhance the use of existing brain banks, allowing us to study several somatic and psychiatric disorders and get a better understanding of the aetiologies.

## Supplementary Information

Below is the link to the electronic supplementary material.

ESM 1 (DOCX 3.53 MB)

## Data Availability

The data is available upon reasonable request.
