# Supplementary material for: Mitigating Fixation Artifacts in Spatial Transcriptomics: Methodological Insights from Human Brain Tissue
Source: Mol Neurobiol. 2026 Jul 15;63(1):765. doi: 10.1007/s12035-026-06033-1 (PMC13372838; doi:10.1007/s12035-026-06033-1)
Supplement: Supplementary file 1 — (DOCX 3.53 MB) [file 12035_2026_6033_MOESM1_ESM.docx]

# **Supplementary Information**

# Mitigating Fixation Artifacts in Spatial Transcriptomics: Methodological Insights from Human Brain Tissue

# ***Molecular Neurobiology***

Kassandra Georges*, Maiken Krogsbaek, Sara Newell Jensen, Thomas Damm Als, Kasper Thorsen, Johannes Rødbro Busch, Jytte Banner, Jens Randel Nyengaard.

*[kasgeo@biomed.au.dk](mailto:kasgeo@biomed.au.dk), Core Center for Molecular Morphology, Department of Clinical Medicine, Aarhus University, Aarhus, Denmark

### **Supplementary File 1** Immunohistochemistry

The tissue was removed from the -80 °C freezer and rehydrated in phosphate-buffered saline solution with Triton X (0.5%) (PBS-T) twice for five minutes. Antigen retrieval was performed in sodium citrate (pH 6.0, 2.94 g in 1 L dH2O) for 15 minutes in a water bath at 85 °C. The tissue was washed in PBS-T for five minutes twice, and subsequently in PBS for five minutes twice. To reduce background signalling, the tissue was quenched by adding TrueBlack diluted 20x in ethanol (70%) for three minutes and rinsed in PBS again twice. The sections were blocked with 1% skim milk powder in PBS for an hour at room temperature (RT), and then incubated with the primary antibody Corticotropin-Releasing Hormone (CRH) 1:4000 - T4037 (rabbit) (BMA Biomedicals) overnight at 4 °C. The following day, sections were rinsed four times for five minutes in PBS and were incubated with secondary antibody Alexa Fluor 568 anti-rabbit IgG 1:400 - A11011 (goat) (0.02 mg/mL, Invitrogen) for an hour at RT. The slides were rinsed in PBS for five minutes four times and afterwards incubated with DAPI (Invitrogen, 0.1 µg/mL) for another five minutes. Lastly, the coverslips were mounted onto the slides with fluorescent mounting medium (DAKO). Images were captured with the NanoZoomer S60 digital slide scanner (C13210-04, Hamamatsu) through the software Nanozoomer Digital Pathology-Scan (NDP.scan). The filters TxRed-Red6 at 160 ms and DAPI at 48 ms were used. Images were taken at 20x magnification.

The sections were analysed in the programme Visiopharm. Through the programme, AI-driven Precision Pathology (APPs) were created to measure following values: The number of CRH-immunoreactive neuron profiles within the PVN; the intensity of the fluorescence from the CRH-immunoreactive neurons (nuclei + soma) within the PVN through the TxRed-Red6 filter; the total area of the PVN; the area of CRH-immunoreactive neurons within the PVN; and lastly the numerical density of the CRH-immunoreactive neurons within the PVN. To avoid the APP misinterpreting artefacts, such as blood with high autofluorescence, as CRH^+^ neurons, a measurement of the maximum intensity was made on control neighbouring sections without any added primary or secondary antibodies and used as a threshold for the minimum intensity. Additional processes were added, including exclusion of nuclei label with an area smaller than 19 µm^2^ or larger than 250 µm^2^; exclusion if the major axis was above 20 µm and under 5 µm, to exclude blood cells; exclusion of CRH label if the area was larger than 500 µm. The post-process included nuclei that were surrounded by CRH staining by 15% and with a distance between nuclei label and CRH label of no more than 2 µm. PVN was outlined manually by guidance from previous Nissl- and CRH stainings. The developed APP was tested on several sections from different patients to ensure a uniform APP that could be applied on all patients, independent of the diagnosis, fixation time or other factors that may play a role. For numerous cases, the APP was compared to the results from a manual count performed previously to ensure that the same amount of CRH^+^ neurons were counted.

### **Supplementary File 2** Spatial transcriptomics protocol

The experiment was performed following the standard Nanostring protocol for fixed frozen tissue with some modifications. Selection of sections containing the paraventricular nucleus of the hypothalamus (PVN) was guided by immunohistochemical staining of corticotropin releasing hormone (CRH) in one full series from each hypothalamus. The full protocol is below. Each section was evaluated on CRH^+^ neuron presence and the section with the highest prevalence of CRH neurons was chosen from each hypothalamus.

For pre-preparation, slides containing 10 µm thick hypothalamus sections were briefly rinsed in PBS, prior to baking at 60^o^C for one hour in a hybridization oven (HybEZ II Oven, ACD Bio). The tissue was shortly washed in 50%, 70%, and two times 100% EtOH. The slides were quickly submerged in 85 °C diethyl pyrocarbonate (DEPC) treated water (BN-51100, BioNordika), before performing target retrieval with 10x Tris-ethylenediaminetetraacetic acid (Tris-EDTA) (00-4956-58, Invitrogen) at 85 °C for 15 minutes for trial 1 and 99 °C for 30 minutes for trial 2. The sections were briefly washed in PBS, prior to incubation with Proteinase K (0.1 µg/mL in PBS, 25530049, Invitrogen) for 15 minutes at 37^o^C. Before moving on to in situ hybridization (ISH), the sections were rinsed in PBS twice. ISH was performed by adding 200 µL of hybridization solution consisting of GeoMx Human Whole Transcriptome Atlas (GMX-RNA-NGS-HuWTA-4, HWTA21004, Nanostring), Buffer R (Nanostring) and DEPC-treated water to each slide. The slides were covered with HydriSlip (GBL714022-100EA, Grace Bio-Labs) before being incubated overnight at 37^o^C in the hybridization oven. The following day, the slides were briefly submerged in 2X Saline Sodium Citrate (SSC) buffer (SIGMA), before performing two stringent washes, consisting of 4X SSC and 100% formamide (AppliChem) at 1:1, at 37^o^C for 25 minutes each. After the stringent washes, the slides were shortly washed twice in 2X SSC. The tissue was blocked for 30 minutes with 200 µL buffer W (Nanostring) per slide, before incubating the slides for one hour in a humidity chamber with the primary antibody against CRH neurons (Rabbit anti-CRH, T4037, BMA Biomedicals, 1:1000) in buffer W. The sections were briefly washed three times in 2xSSC, before 30 minutes incubation with the secondary antibody (Alexa Fluor 594 goat anti-Rabbit IgG, 2 mg/ml, A-11012, Invitrogen), followed by another three washes in 2xSSC. The sections were incubated with an isotype control (IgG Rabbit, ab37415, Abcam) for 30 minutes, followed by three additional washes in 2xSSC. The nuclei stain SYTO83 (S11364, Invitrogen) was used for further incubation of the sections, followed by three times wash in 2xSSC. Afterwards the slides were incubated for an hour with the primary antibody against microglia, Iba1 (Rabbit anti-Iba1, 019-19741, Wako, 1:1000) and then received another round of three washes in 2xSSC. Lastly, the sections were incubated with a secondary antibody (Alexa Fluor 488 goat anti-rabbit IgG, 5 µg/ml, A11034, Invitrogen) and washed in three times 2xSSC before being loaded onto the GeoMx DSP instrument.

Images of the sections were captured with a 20x objective (NA = 0.45), and based on these, two to four regions of interest (ROIs) with dimensions of maximum 660 µm x 785 µm were localized along the PVN for each patient. Within every ROI, two sub-regions with biological targets, also known as area of illumination (AOI) were made, one for CRH^+^ neurons and one for Iba1^+^ microglia. The segment identifying CRH^+^ neurons and the segment identifying Iba1^+^ microglia had following characteristics, respectively: Erode 1 µm, N-Dilate 2 µm, Hole Size 160 µm^2^, Particle size 15 µm^2^; Erode 1 µm, N-Dilate 2 µm, Hole Size 1 µm^2^, Particle size 10 µm^2^. For the individual ROIs, the threshold sensitivity for each channel was modified to ensure coverage of all cells of interest. The barcodes from the CRH^+^ neuron segments were cleaved off and collected in a 96-well collection plate first, and the Iba1^+^ microglia segments second.

The collection plate was prepared for readout by NGS by performing Polymerase Chain Reaction (PCR) amplification. The collection plate was covered with a permeable membrane and incubated on a thermocycler at 65 °C with the lid open for one hour to evaporate residual liquid. Next the permeable membrane was replaced with a PCR seal and was subsequently spun down. By adding 10 µL of nuclease-free water to the individual well at RT for 10 minutes, the targets were rehydrated, and a new adhesive plate seal was added. Plates for SeqCode Primer and 5X PCR Master Mix were thawed and centrifuged to 1000 x *g* alongside the collection plate. PCR amplification could then take place in a new PCR plate by firstly adding 2 µL of 5X PCR Master Mix at the bottom of each well. An additional 4 µL of the SeqCode Primer was added to the PCR plate. Finally, 4 µL of the DSP barcodes, including the no-template control (NTC), from the original collection plate was added to the corresponding wells in the PCR plate. This was followed by pipetting the wells 10 times, sealing the plate with a PCR plate sticker, pulse centrifuging the plate to 1000 x *g*, and incubating the plate in a thermocycler. The PCR plate was processed according to the specified PCR program. Following completion of the PCR programme, the library was subjected to AMPure cleanup. First the PCR plate was centrifuged to 1000 x *g*, and 4 µL of each PCR product was pooled into the same 1.5 mL tube, including NTC, but excluding the PCR positive control. For the NTC PCR product, additional 6 µL were pipetted into another 1.5 mL tube. A third 1.5 mL tube was filled with 10 µL PCR positive control. AMPure XP beads were added to each tube at the appropriate ratio, mixed, centrifuged, and incubated for 5 minutes at room temperature. The supernatant was removed by pelleting the beads on a magnetic stand for five minutes. Two rounds of washing followed, by adding 1 mL of EtOH (80%) to the tube, and the supernatant was repeatedly removed after 30 seconds on the magnetic stand. The beads were left to dry for five minutes on the magnetic stand and were then resuspended in 54 µL Elution Buffer for five minutes at RT followed by additional five minutes on the magnetic stand to form pellets. 50 µL of supernatant were transferred to a new tube and 60 µL AMPure XP beads were added, mixed by pipetting, and pulse centrifuged. After a 5-minute incubation at room temperature, beads were pelleted on a magnetic stand, washed twice with 1 mL of 80% ethanol, and air-dried for up to 5 minutes. Beads were then resuspended in 16 µL of Elution Buffer. The quality of the library stock and its dilution, including the purified NTC, was assessed using an Agilent TapeStation. Library stock concentration was then quantified with a Qubit fluorometer. Libraries were loaded at a final concentration of 250 pM onto an Illumina NovaSeq 6000 platform using an SP flow cell.

After sequencing raw reads in the form of FASTQ files were generated. By using the GeoMx NGS Pipeline (v2.3.3.10) through NanoString’s standalone software, the FASTQ files could be converted to digital count conversion (DCC). Here, the FASTQ files were processed, including removing the adaptor sequences computationally and merging the overlapping paired ends, resulting in the stitched reads. By aligning these stitched reads to the RTS-ID barcodes, aligned reads were created. Raw counts were assigned to their biological target. The unique reads in the DCC files were identified by removing PCR duplicates based on their UMI sequence in each read.

For GeoMx data quality control DCC files were uploaded into the GeoMx DSP platform, and quality control procedures were performed. Read trimming, stitching, and alignment percentages were evaluated alongside sequencing saturation. Although a few AOIs had trimming rates exceeding 20%, they were retained for analysis as other quality metrics remained acceptable. All NTC counts were below 5,000, and sequencing saturation was greater than 94% for all AOIs. Due to the small size of ROIs and the sparsity of cells, AOI size and nuclei counts were generally low. Nevertheless, each AOI generated more than 1.4 million raw reads, with >7,500 deduplicated reads in trial 1 and >40,000 deduplicated reads in trial 2.

**Supplementary Table 1** Summary statistics of of human subjects

| **Variable** | **Value** |
| --- | --- |
| Number of subjects | 20 |
| Age, years | 55.0 (43.3-70.0) |
| Sex, n (%) | Female: 9 (45%) |
|  | Male: 11 (55%) |
| BMI | 28.2 (24.7-31.3) |
| PMI, hr | 116.1 (90.9-148.5) |
| Fixation period, days | 307.0 (102.8-547.3) |
| *Footnote: Continuous variables are presented as median (interquartile range)* | |

**Supplementary Table 2** ACD Bio RNAscope scoring criteria [1]


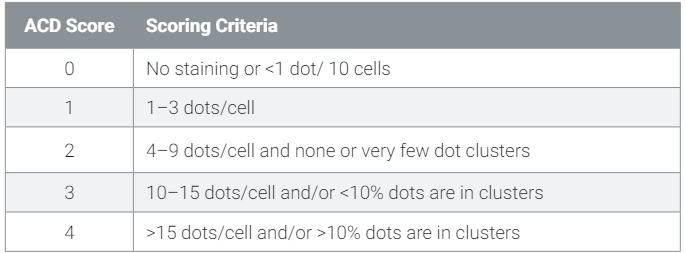


**Supplementary Table 3** GeoMx DSP: Nuclei count/AOI for trial 1 and 2

| **Trial_Section** | **Fixation time (days)** | **Nuclei Count in AOI** |
| --- | --- | --- |
| Trial-1_S1 | 34 | 47 |
| Trial-1_S1 | 34 | 55 |
| Trial-1_S1 | 34 | 27 |
| Trial-1_S1 | 34 | 33 |
| Trial-1_S3 | 477 | 41 |
| Trial-1_S3 | 477 | 71 |
| Trial-1_S3 | 477 | 43 |
| Trial-1_S3 | 477 | 44 |
| Trial-1_S3 | 477 | 40 |
| Trial-1_S4 | 751 | 188 |
| Trial-1_S4 | 751 | 79 |
| Trial-1_S4 | 751 | 87 |
| Trial-1_S4 | 751 | 73 |
| Trial-1_S4 | 751 | 53 |
| Trial-2_S1 | 34 | 79 |
| Trial-2_S1 | 34 | 92 |
| Trial-2_S1 | 34 | 61 |
| Trial-2_S3 | 477 | 48 |
| Trial-2_S3 | 477 | 8 |
| Trial-2_S3 | 477 | 35 |
| Trial-2_S4 | 751 | 78 |
| Trial-2_S4 | 751 | 87 |
| Trial-2_S4 | 751 | 68 |

**Supplementary Table 4** GeoMx DSP: Raw reads/µm^2^ for trial 1 and 2

| **Trial_Section** | **Raw reads/µm^2^** |
| --- | --- |
| Trial-1_S1 | 2151 |
| Trial-1_S1 | 1021 |
| Trial-1_S1 | 2692 |
| Trial-1_S1 | 1521 |
| Trial-1_S3 | 2396 |
| Trial-1_S3 | 977 |
| Trial-1_S3 | 481 |
| Trial-1_S3 | 1555 |
| Trial-1_S3 | 711 |
| Trial-1_S4 | 514 |
| Trial-1_S4 | 302 |
| Trial-1_S4 | 637 |
| Trial-1_S4 | 565 |
| Trial-1_S4 | 2304 |
| Trial-2_S1 | 822 |
| Trial-2_S1 | 1362 |
| Trial-2_S1 | 1418 |
| Trial-2_S3 | 732 |
| Trial-2_S3 | 1088 |
| Trial-2_S3 | 893 |
| Trial-2_S4 | 323 |
| Trial-2_S4 | 379 |
| Trial-2_S4 | 395 |

**Supplementary Table 5** GeoMx DSP: AOI Surface Area (µm^2^) for trial 1 and 2

| **Trial_Section** | **AOI Surface Area (µm^2^)** |
| --- | --- |
| Trial-1_S1 | 4210 |
| Trial-1_S1 | 4499 |
| Trial-1_S1 | 2154 |
| Trial-1_S1 | 2377 |
| Trial-1_S3 | 2936 |
| Trial-1_S3 | 4228 |
| Trial-1_S3 | 3305 |
| Trial-1_S3 | 3668 |
| Trial-1_S3 | 2787 |
| Trial-1_S4 | 9300 |
| Trial-1_S4 | 5613 |
| Trial-1_S4 | 4963 |
| Trial-1_S4 | 3860 |
| Trial-1_S4 | 2859 |
| Trial-2_S1 | 7924 |
| Trial-2_S1 | 7727 |
| Trial-2_S1 | 5627 |
| Trial-2_S3 | 5185 |
| Trial-2_S3 | 4716 |
| Trial-2_S3 | 4832 |
| Trial-2_S4 | 5285 |
| Trial-2_S4 | 5971 |
| Trial-2_S4 | 5000 |

**Supplementary Fig. 1** Regions of interests along the paraventricular nucleus of the hypothalamus for spatial transcriptomics


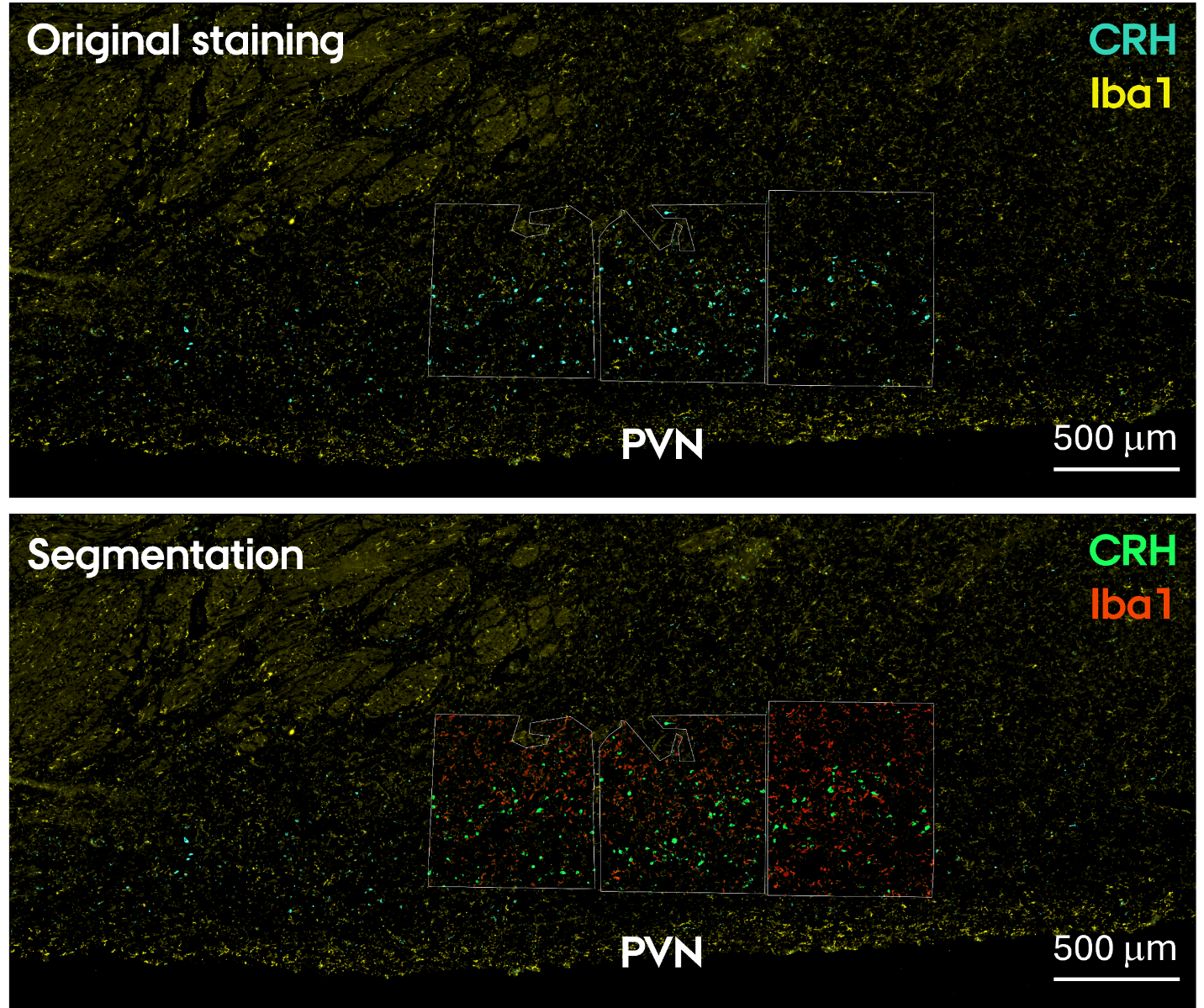


***Supplementary Fig. 1*** *Example of three regions of interests (ROIs) along the paraventricular nucleus of the hypothalamus (PVN). Top: Original staining of CRH and Iba1. Bottom: the original staining of CRH and Iba1 with their individual segmentation masks overlayed within the ROIs.*

**Supplementary Fig. 2** RNAscope manual evaluation score

***Supplementary Fig. 2*** *Manual evaluation score of RNAscope experiment on the paraventricular nucleus of the hypothalamus (PVN) on four different fixation periods (34, 112, 477, and 751 days) and following 5, 15, and 30 minutes target retrieval*

**Supplementary Fig. 3** Comparison of ACD score between manual evaluation and QuPath quantification

|  | **Automatic** | | | | | |  | **Manual** | | |  |  |  |  |
| --- | --- | --- | --- | --- | --- | --- | --- | --- | --- | --- | --- | --- | --- | --- |
| **Fixation time (days)** | **5** | | **15** | | **30** | |  | **5** | **15** | **30** |  | ACD-score | | |
| **34** | 0 | 1 | 4 | 4 | 4 | 4 |  | 0 | 4 | 4 |  |  | 1 |  |
| **112** | 0 | 0 | 1 | 4 | 4 | 4 |  | 0 | 3 | 4 |  |  | 2 |  |
| **477** | 0 | 0 | 0 | 0 | 4 | 4 |  | 0 | 0 | 4 |  |  | 3 |  |
| **751** | 0 | 0 | 0 | 0 | 1 | 4 |  | 0 | 0 | 2 |  |  | 4 |  |
|  | PVN | ARC | PVN | ARC | PVN | ARC |  | Whole section | | |  |  |  |  |

***Supplementary Fig. 3*** *Comparison of ACD score between manual evaluation and QuPath quantification. Target retrieval duration: 5, 15, 30 min. Paraventricular nucleus, PVN; arcuate nucleus, ARC*

**Supplementary Fig. 4** StandR data analysis Quality Control

Explained variance (%)

| **A) QC Gene level** | **B) QC ROI level: AOI nuclei count** |
| --- | --- |
| 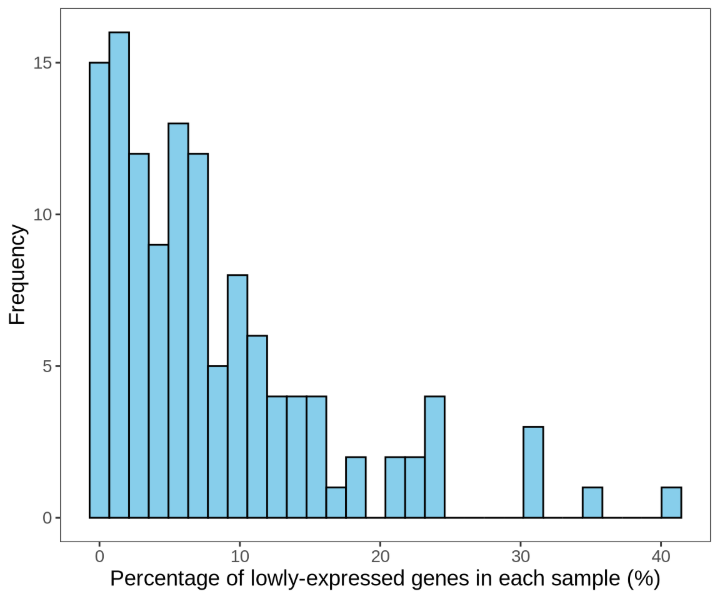 | **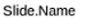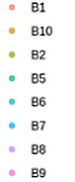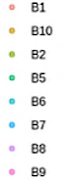**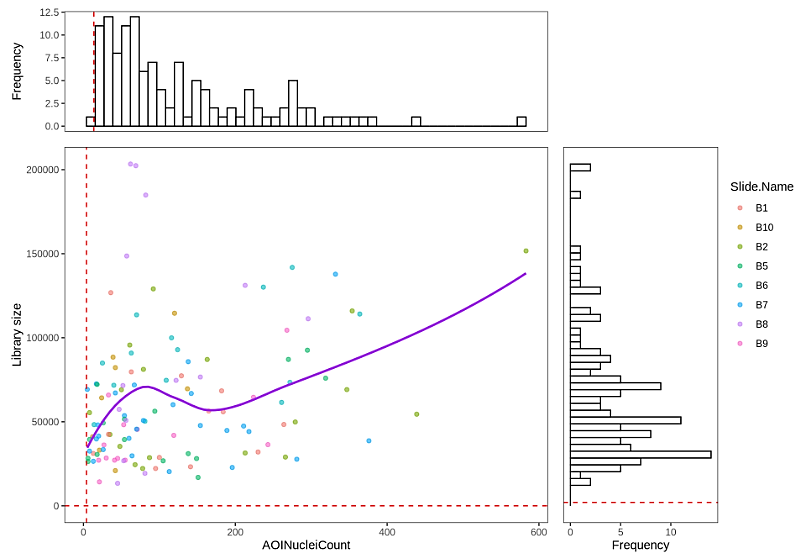 |
|  |  |
| **D) PCA Scree plot** | **C) QC ROI level: AOI size** |
| 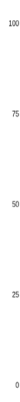  **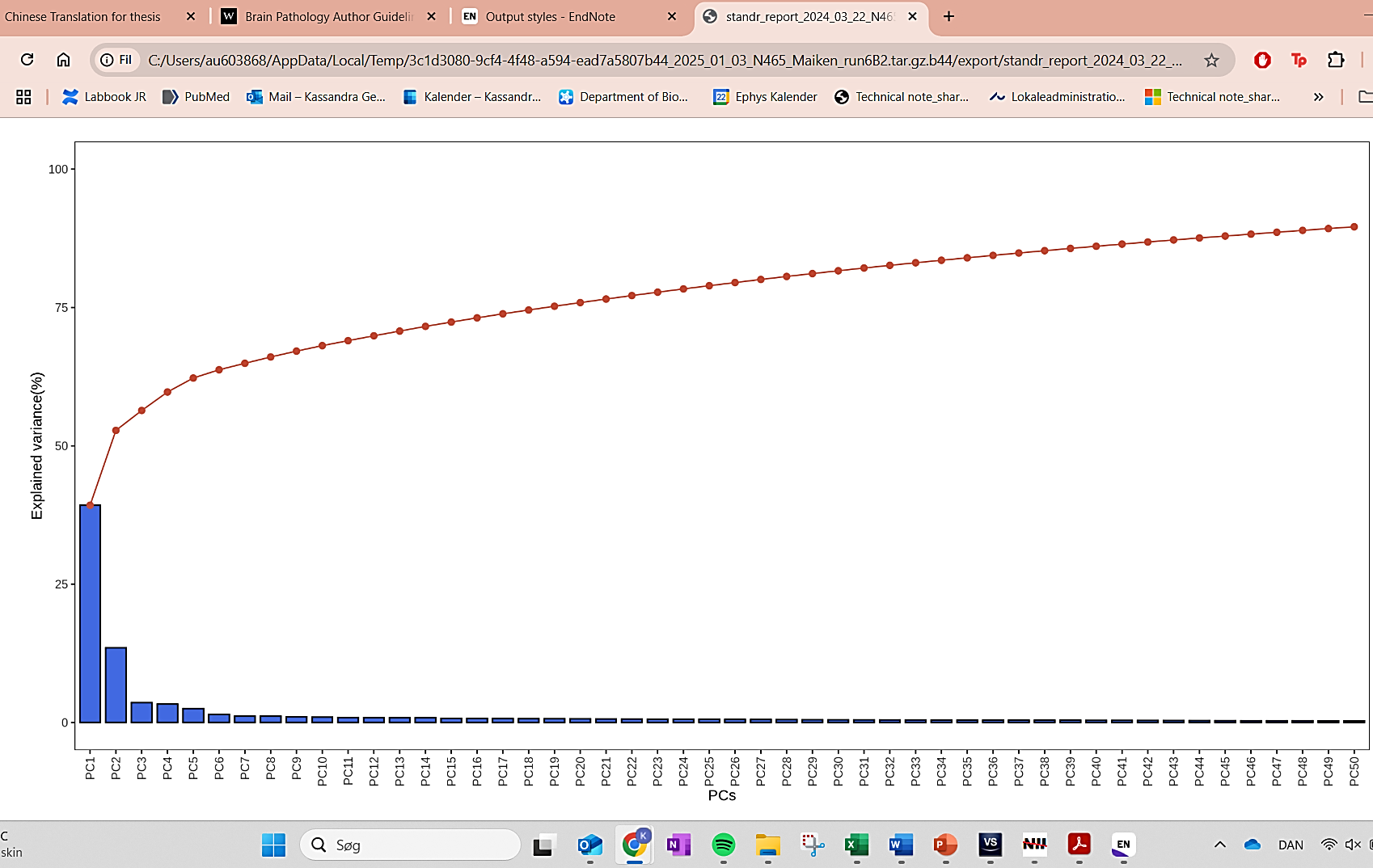**  PCs | 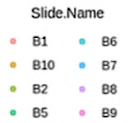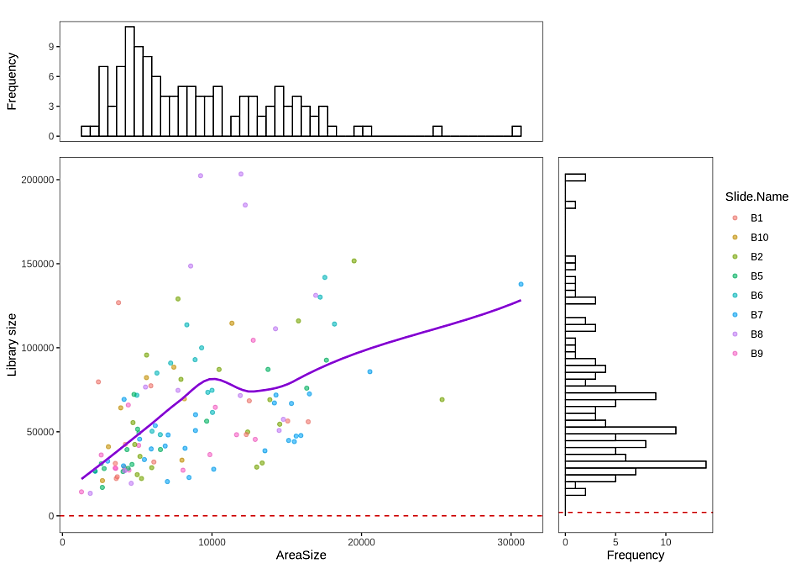 |

**Supplementary Fig. 4** A selection of StandR data analysis quality control (QC) parameters, including: (A) Gene level QC, (B) ROI level QC AOI nuclei count, (C) ROI level QC AOI size, (D) PCA scree plot. Area of illumination, AOI; Relative log expression, RLE

**Supplementary Fig. 5** PCA plots before and after RUV4 batch correction with $k\in\left[ 0:5 \right]$

***Supplementary Fig. 5*** *PCA plots before and after RUV4 batch correction with k∈[0:5]. PCA plots including factors of biological target, slide, body mass index (BMI), age, section, post-mortem interval (PMI), fixed period, and grouped fixation time.*

## References

1. Advanced Cell Diagnostics. *A Guide for RNAscope® Data Analysis - a Wealth of Gene Expression Information from Tissue Context*. 2017; Available from: <https://fab.cba.mit.edu/classes/S63.21/class_site/pages/class_5/Supplemental%20PDFS/MK_51_103_RNAScope_data_analysis_guide_RevB.pdf>.
